# Manipulating Copper Dispersion on Ceria for Enhanced Catalysis: A Nanocrystal‐Based Atom‐Trapping Strategy

**DOI:** 10.1002/advs.202104749

**Published:** 2022-01-20

**Authors:** Yifan Sun, Felipe Polo‐Garzon, Zhenghong Bao, Jisue Moon, Zhennan Huang, Hao Chen, Zitao Chen, Zhenzhen Yang, Miaofang Chi, Zili Wu, Jue Liu, Sheng Dai

**Affiliations:** ^1^ Chemical Sciences Division Oak Ridge National Laboratory Oak Ridge TN 37831 USA; ^2^ Center for Nanophase Materials Sciences Oak Ridge National Laboratory Oak Ridge TN 37831 USA; ^3^ Department of Chemistry The University of Tennessee Knoxville TN 37996 USA; ^4^ Neutron Scattering Division Oak Ridge National Laboratory Oak Ridge TN 37831 USA

**Keywords:** atom‐trapping, colloidal nanocrystal, copper‐ceria, water–gas shift reaction

## Abstract

Due to tunable redox properties and cost‐effectiveness, copper‐ceria (Cu‐CeO_2_) materials have been investigated for a wide scope of catalytic reactions. However, accurately identifying and rationally tuning the local structures in Cu‐CeO_2_ have remained challenging, especially for nanomaterials with inherent structural complexities involving surfaces, interfaces, and defects. Here, a nanocrystal‐based atom‐trapping strategy to access atomically precise Cu‐CeO_2_ nanostructures for enhanced catalysis is reported. Driven by the interfacial interactions between the presynthesized Cu and CeO_2_ nanocrystals, Cu atoms migrate and redisperse onto the CeO_2_ surface via a solid–solid route. This interfacial restructuring behavior facilitates tuning of the copper dispersion and the associated creation of surface oxygen defects on CeO_2_, which gives rise to enhanced activities and stabilities catalyzing water–gas shift reaction. Combining soft and solid‐state chemistry of colloidal nanocrystals provide a well‐defined platform to understand, elucidate, and harness metal–support interactions. The dynamic behavior of the supported metal species can be further exploited to realize exquisite control and rational design of multicomponent nanocatalysts.

## Graphical abstract

Understanding and harnessing the restructuring of nanocrystals is important for catalytic applications, while elucidating the surface and bulk characteristics remains challenging. A nanocrystal‐based atom‐trapping strategy is reported to access well‐defined copper‐ceria nanostructures with tunable copper dispersions. The optimized copper‐ceria nanocatalysts exhibit enhanced activities and stabilities catalyzing water−gas shift reaction.

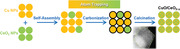

## 1 Introduction

Supported metal nanostructures are an important class of materials for catalysis. Ranging from single atoms to subnanomeric clusters and nanocrystals, the geometric and electronic properties of supported metal catalysts can be exquisitely tailored through modulating the interactions between the supported metals and the underlying substrate.^[^ ^1^ ^]^ For example, recent advances in single‐atom catalysts have brought unprecedented opportunities to unveil the bonding, coordination, and charge transfer at the atomic level that influence catalytic performances.^[^ ^2^ ^]^ Unlike noble metals such as Au and Pd that form discrete supported domains, transition metals tend to encompass more dynamic structural transformations.^[^ ^3^ ^]^ The higher intrinsic reactivities of transition metals cause atom migration, lattice substitution, and interfacial restructuring under synthesis and reaction conditions, which makes it challenging to elucidate and modify the local chemical environments.^[^ ^4^ ^]^

With tunable properties and low cost, copper‐ceria (Cu‐CeO_2_) materials have been applied to catalyze a wide scope of reactions such as CO and hydrocarbon oxidation,^[^ ^5^, ^6^, ^7^ ^]^ water*–*gas shift reaction (WGSR),^[^ ^8^, ^9^ ^]^ CO_2_ hydrogenation to methanol,^[^ ^10^, ^11^ ^]^ and multiple electrocatalytic and photocatalytic processes.^[^ ^12^, ^13^ ^]^ Despite the historical development, building well‐defined Cu‐CeO_2_ nanocatalysts has remained elusive. Ceria is structurally robust as substrates, while incorporation of aliovalent copper promotes generation of oxygen defects. Copper, on the other hand, is amenable to reformation at elevated temperatures, forming segregated oxide phases or substituting into the oxide lattice of ceria.^[^ ^14^ ^]^ From a synthesis perspective, the interplay between Cu and CeO_2_ domains plays a decisive role in tuning the local structures in Cu‐CeO_2_. Parameters including shape, morphology, and size of CeO_2_, loading density and dispersion of Cu, as well as surface area and porosity, all have significant influences on the catalytic properties of Cu‐CeO_2_.^[^ ^15^ ^]^ The inherent structure complexities involving surface, interface, and bulk domains also induce inconsistencies for mechanistic studies based on samples prepared using different methods. It is thereby greatly desired to develop generalizable and robust synthesis protocols to access precise Cu‐CeO_2_ nanostructures for catalysis.

Governed by soft and solid‐state chemistry, colloidal nanocrystals can function as precursors and intermediates to fabricate multinary metal oxides and sulfides with adjustable compositions and sizes.^[^ ^16^ ^]^ The higher reactivities of nanoscale solids relative to the bulk counterparts also facilitate structure tuning under mild conditions. Prior studies on nanocrystal‐based solid‐state synthesis has mainly focused on 2D, substrate‐confined architectures for electro‐ and photocatalysis.^[^ ^17^, ^18^, ^19^ ^]^ By contrast, 3D, freestanding powder samples derived from nanocrystal precursors, which benefits charge and mass transport for thermocatalysis, have been underexplored. Here, we report a nanocrystal‐based atom‐trapping strategy to access well‐defined Cu‐CeO_2_ nanostructures for thermocatalysis. Nanostructured CuO/CeO_2−_ *_x_* samples were prepared through controlled self‐assembly and interface restructuring of colloidal Cu and CeO_2_ nanoparticles. Combining a suite of characterization techniques, we demonstrate that Cu tend to be atomically redispersed and stabilized onto the surface of the CeO_2_ nanocrystals. This synthesis strategy was further extended to the preparation of Cu/CeO_2_ nanocatalysts, where optimized Cu dispersion gives rise to enhanced activities and stabilities catalyzing WGSR.

## 2 Results and Discussion

### 2.1 Restructuring of Cu Nanocrystals on CeO_2_

**Figure 1:**
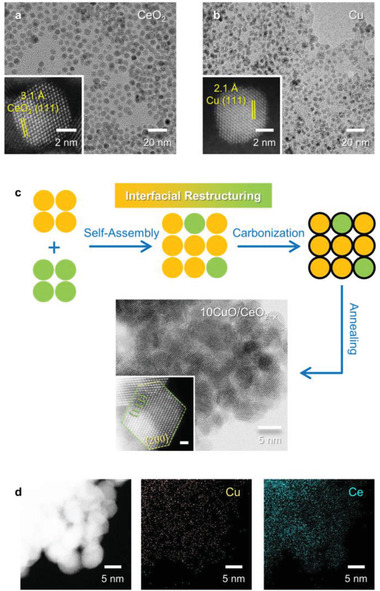
TEM images of colloidal a) CeO_2_ and b) Cu nanocrystals with the inset showing the corresponding high‐resolution HAADF‐STEM images. c) Schematic illustrating the interfacial restructuring strategy, and the HAABF‐STEM image of the calcinated 10CuO/CeO_2−_ *_x_* nanopowders. The inset displays the high‐resolution HAADF‐STEM image of the 10CuO/CeO_2−_ *_x_* nanocrystals with the well‐retained truncated octahedral shape upon calcination (scale bar: 1 nm). The {111} and {200} facet is highlighted using the green and yellow dotted lines, respectively. d) HAADF‐STEM image with the corresponding EDS element maps of 10CuO/CeO_2−_ *_x_*.

As reaction precursors, monodisperse and single‐crystalline Cu (4.8±0.6 nm) and CeO_2_ nanocrystals (5.9±0.7 nm) were individually prepared using colloidal synthesis,^[^ ^20^, ^21^ ^]^ as shown by the transmission electron microscopy (TEM) images in **Figure** **1**a,b; and Figure S1a (Supporting Information). A solution‐based, ligand‐directed self‐assembly method was applied to the colloidal mixture of Cu (10 at%) and of CeO_2_ (90 at%),^[^ ^22^, ^23^ ^]^ with the aim to increase the interfacial area between the Cu and CeO_2_ domains (Figure S1b, Supporting Information). Annealing under inert atmospheres caused carbonization of the long‐chain organic ligands with enhanced metal–support interactions.^[^ ^24^ ^]^ Further calcination in air removed the carbon shell and yielded mesoporous aggregates of nanocrystals, as evidenced by the high‐angle annular bright‐field scanning transmission electron microscopy (HAABF‐STEM) image in Figure 1c. The obtained powder samples were denoted as 10CuO/CeO_2−_ *_x_* to highlight formation of the heterostructured interface. The inductively coupled plasma‐atomic emission spectroscopy (ICP‐AES) results (Table S1, Supporting Information) confirmed around 10 mol% of Cu is retained for 10CuO/CeO_2−_ *_x_*. Conventional fabrication approaches, such as deposition‐precipitation, coprecipitation, and impregnation methods, typically rely on delicate control of the experimental parameters including temperature, time, concentration, and pH of the solution to adjust the Cu content in the Cu‐CeO_2_ samples.^[^ ^8^, ^9^, ^25^ ^]^ In comparison, the stoichiometric process demonstrated here facilities better control for composition tuning in a stoichiometric manner.

**Figure 2:**
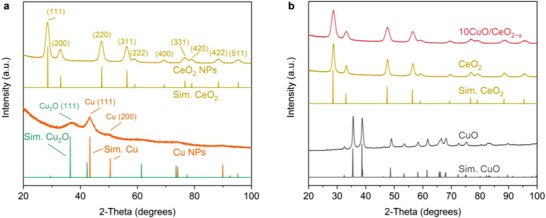
Powder XRD patterns for a) the colloidal Cu and CeO_2_ nanocrystals, and b) the calcinated CeO_2_ and 10CuO/CeO_2−_ *_x_* samples. Simulated diffraction patterns based on the crystal structures of bulk CeO_2_, Cu, Cu_2_O, and CuO are provided for comparison.^[^ ^26^, ^27^, ^28^ ^]^

**Figure** **2**a shows the powder X‐ray diffraction (XRD) pattern of the as‐synthesized pristine Cu and CeO_2_ nanocrystals, as well as the corresponding references.^[^ ^26^, ^27^, ^28^ ^]^ The colloidal Cu nanoparticles displayed two broad peaks corresponding to the (111) and (200) planes of Cu, together with a noticeable peak assigned to the (111) peak of Cu_2_O due to rapid surface oxidation. The colloidal CeO_2_ nanocrystals exhibited characteristic crystalline peaks corresponding to the fluorite structure of CeO_2_. Despite the observed differences for the colloidal nanocrystal precursors, the calcinated, ligand‐free CuO/CeO_2−_ *_x_* sample showed nearly identical XRD patterns compared with that of CeO_2_ (Figure 2b). Absence of the diffraction peaks corresponding to the CuO phase indicates that the Cu nanoparticles underwent restructuring upon oxidation on CeO_2_ instead of topological transformations.^[^ ^29^ ^]^ This is further supported by the high‐angle annular dark‐field scanning transmission electron microscopy (HAADF‐STEM) images with corresponding STEM‐energy dispersive X‐ray spectroscopy (EDS) element maps, showing a homogeneous distribution of the Cu signal throughout the CeO_2_ domain (Figure 1d). The truncated octahedral shape terminated with the {111} and {001} facets of the CeO_2_ domain was also well retained (Figure 1c), and no crystalline CuO phase was observed upon calcination.

**Figure 3:**
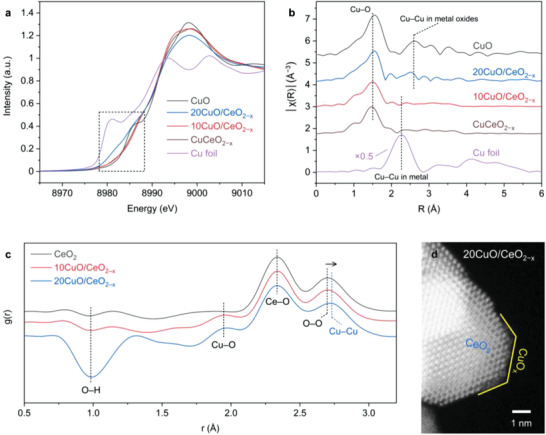
a) Normalized Cu K‐edge XANES spectra highlighting the pre‐edge region and b) EXAFS spectra of the CuO/CeO_2−_ *_x_* and CuCeO_2−_ *_x_* samples with Cu and CuO references. c) Neutron pair distribution function g(r) data for CeO_2_, 10CuO/CeO_2−_ *_x_*, and 20 CuO/CeO_2−_ *_x_*. d) High‐resolution image capturing the CuO*_x_* atomic clusters (yellow line) supported on the CeO_2_ nanocrystals.

To shed light on the restructuring behavior of the Cu nanocrystals, X‐ray adsorption spectroscopy (XAS) and neutron diffraction were conducted. Samples including 20CuO/CeO_2−_ *_x_* with higher Cu loading densities, and copper‐substituted ceria nanopowders (CuCeO_2−_ *_x_*),^[^ ^30^ ^]^ were also prepared for comparison (Figure S2, Supporting Information). The Cu K‐edge X‐ray absorption near edge structure (XANES) spectra in **Figure** **3**a reveals the prevalence of Cu (II) in 10CuO/CeO_2−_ *_x_*, 20CuO/CeO_2−_ *_x_*, and CuCeO_2−_ *_x_*.^[^ ^14^, ^31^, ^32^ ^]^ Figure 3b displays the corresponding Fourier‐transformed extended X‐ray absorption fine structure (EXAFS) spectra. Interestingly, an almost identical tricoordinated Cu_1_O_3_ geometry was observed for the Cu—O bond in 10CuO/CeO_2−_ *_x_* (R ≈ 1.95 Å and CN ≈ 3.1) and CuCeO_2−_ *_x_* (R ≈ 1.94 Å and CN ≈ 3.3), with the fitting results summarized in Figure S3 and Table S2 (Supporting Information).^[^ ^32^ ^]^ Peaks corresponding to scattering beyond the first coordination sphere were not observed, revealing the atomic dispersion of copper species in both 10CuO/CeO_2−_ *_x_* and CuCeO_2−_ *_x_*. Besides the ideal tri‐coordination, the CN of Cu can be higher than 3 with different substitutional and nonsubstitutional structure models,^[^ ^14^, ^30^, ^32^ ^]^ and distinct underlying facets of CeO_2_ ({111} versus {200}) as shown in Figure 1c. Increasing the Cu content resulted in formation of aggregated CuO*_x_* clusters, manifested by the emergence of the Cu—Cu contribution (R ≈ 2.93 Å and CN ≈ 0.9) in addition to the first‐shell Cu—O bond (R ≈ 1.95 Å and CN ≈ 3.1) for 20CuO/CeO_2−_ *_x_*. Therefore, despite distinct synthesis pathways for 10CuO/CeO_2−_ *_x_* and CuCeO_2−_ *_x_* that involve different reactants and intermediates, they share the same destination where copper atoms are stabilized in the fluorite lattice of CeO_2_ as cationic substituents.

Neutron diffraction was employed to probe the fluorite structure in the CeO_2_, 10CuO/CeO_2−_ *_x_*, and 20CuO/CeO_2−_ *_x_* samples, which helps identify the spatial distribution of the copper species. The obtained diffraction data were interpreted in real space using pair distribution function (PDF) analysis, which utilizes both Bragg and diffuse scattering information and thereby possesses high sensitivities to short‐ and intermediate‐range structures in nanoscale and amorphous systems.^[^ ^33^, ^34^ ^]^ The short‐ and intermediate‐range g(r) fittings summarized in Figures S4 and S5 (Supporting Information) imply that Cu incorporation did not alter the lattice parameter of the ceria domain in 10CuO/CeO_2−_ *_x_* and 20CuO/CeO_2−_ *_x_* (a ≈ 5.42 Å) relative to that of the pristine CeO_2_ nanopowders (Tables S3 and S4, Supporting Information). Retention of the bulk fluorite lattice reflects that the copper species tend to become stabilized on the surface rather than diffusing into the bulk lattice of CeO_2_, which is consistent with the low coordination number of Cu in the EXAFS data (Table S2, Supporting Information) and minimal change of the Ce L‐III edge upon copper incorporation (Figure S6, Supporting Information).

In addition, PDF fitting also reveals formation of the phase‐segregated CuO*_x_* clusters in 20CuO/CeO_2−_ *_x_*. A closer look at the short‐range g(r) fitting result brings more insights into the structures in the range of angstroms (Figure 3c). Compared with the pristine CeO_2_ sample, the CuO/CeO_2−_ *_x_* samples showed a distinct negative peak at ≈1.0 Å, which is assigned to the surface‐attached O—H bonds considering the negative coherent nuclear scattering length of hydrogen. Elevation of the Cu loading densities drastically increases the intensity of the negative signal, indicating the CeO_2_‐supported CuO*_x_* surface is more likely to adsorb hydroxyl groups under ambient conditions. Moreover, the peak at ≈2.70 Å corresponding to the O–O contribution for CeO_2_ and 10CuO/CeO_2−_ *_x_* shifts toward longer distances for 20CuO/CeO_2−_ *_x_*. This shift is likely caused by the addition of the minor Cu—Cu contribution (R ≈ 2.93 Å and CN ≈ 0.9) in the phase‐segregated CuO*_x_* clusters.^[^ ^35^ ^]^ The quantitative fit using an additional CuO phase yields noticeably enhanced the fitting results (Figure S4d, Supporting Information). The shift is thus in good agreement with the above EXAFS fitting results (Table S2, Supporting Information). In addition, the subnanomeric CuO*_x_* layers situated on the Cu‐substituted CeO_2_ surface were also captured in the atomic‐resolution HAADF‐STEM images for 20CuO/CeO_2−_ *_x_* (Figure 3d; and Figure S7, Supporting Information), consistent with the bi‐ and tri‐layer copper (oxide) clusters observed by Shen and colleagues.^[^ ^35^ ^]^

The above spectroscopic, diffraction, and microscopic results provide unambiguous evidence showing the dynamic restructuring of the CeO_2_‐supported Cu nanoparticles, which entails detachment from the Cu domains, ripening at the CuO/CeO_2−_ *_x_* interface and redispersion on the CeO_2_ surface in the Cu_1_O_3_ geometry. This process shares the same merit of atom trapping of the volatile Pt species to fabricate the Pt_1_/CeO_2_ catalyst,^[^ ^36^ ^]^ while the solid–solid reactions at lower temperatures permit facile and stoichiometric composition tuning. It can be expected that a wider scope of transition metals besides copper can be reconfigured and incorporated onto the surface of chemically reducible yet structurally robust metal oxides such as CeO_2_ and TiO_2_ using this approach. Structure parameters including size and facet of the supported nanoparticle precursors, and the associated interface and surface effects provide ample opportunities for modulating the local electronic and geometric structures under mild conditions.

### 2.2 Preparation of Composition‐Tunable CuO/CeO_2−__*x*_ Samples

Using this nanoparticle‐based atom‐trapping strategy, we fabricated a series of CuO/CeO_2_ samples that contains 5, 10, 20, and 50 at% of Cu, respectively, which provides a well‐defined platform to manipulate the composition‐dependent dispersion of copper on ceria. As illustrated in Figure S8 (Supporting Information), 5CuO/CeO_2−_ *_x_*, 10CuO/CeO_2−_ *_x_*, and 20CuO/CeO_2−_ *_x_* all exhibit the diffraction pattern of the characteristic fluorite structure, while two peaks corresponding to crystalline CuO phases emerge for 50CuO/CeO_2−_ *_x_*. The CuO*_x_* atomic layers in 20CuO/CeO_2−_ *_x_*, as shown in Figure 3c, can hardly be detected using laboratory XRD due to the limited resolution and broadening of the diffraction peaks for nanoscale solids.^[^ ^37^ ^]^

**Figure 4:**
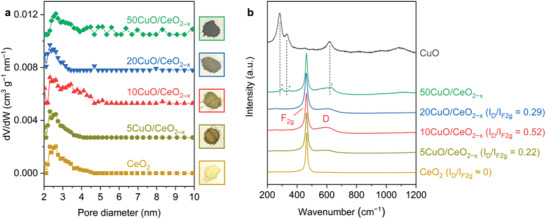
a) Pore‐size distribution and pictures of the CeO_2_ and CuO/CeO_2−_ *_x_* powder samples. b) Raman spectra of the CeO_2_, CuO, and CuO/CeO_2−_ *_x_* samples. The Raman spectra were acquired using a 532 nm excitation laser. Vibration modes corresponding to the segregated CuO*_x_* phase for 50CuO/CeO_2−_ *_x_* were highlighted using asterisks.

The porous structures, which are facilitated by the self‐assembly of the CeO_2_ nanocrystals as building blocks, were investigated using N_2_ adsorption–desorption measurement. As shown in Figure S9a (Supporting Information), introducing copper to the mesoporous architecture of ceria nanocrystals decreases interparticle space and consequently the surface area. The pore‐size distribution in **Figure** **4**a suggests that CeO_2_, 5CuO/CeO_2−_ *_x_*, 10CuO/CeO_2−_ *_x_*, 20CuO/CeO_2−_ *_x_*, as well as CuCeO_2−_ *_x_*, all possess the uniform pore size of 2–4 nm, consistent with the small angle scattering (SAS) results (Figure S9b, Supporting Information). The segregated CuO*_x_* phase in 50CuO/CeO_2−_ *_x_* induces observable pore‐size disorder. Calcinating the assembled colloidal Cu nanoparticles produced bulk CuO with low surface area (14 m^2^ g^−1^), highlighting the high reactivity of the copper nanocrystals as well as the key role of the CeO_2_ support in dispersing the restructured copper species.

Raman spectroscopy was applied to monitor the composition‐dependent structure evolution of the CuO/CeO_2−_ *_x_* samples (Figure 4b). As summarized in Figure S10 (Supporting Information), the F_2g_ mode witnesses a red shift from CeO_2_ (464.7 cm^−1^) to 5CuO/CeO_2−_ *_x_* (463.7 cm^−1^), 10CuO/CeO_2−_ *_x_* (462.9 cm^−1^), and 20CuO/CeO_2−_ *_x_* (461.5 cm^−1^), while blueshifts for 50CuO/CeO_2−_ *_x_* (463.5 cm^−1^). The dispersion of copper is thus strongly correlated with lattice substitution on the surface of ceria.^[^ ^38^ ^]^ 50CuO/CeO_2−_ *_x_* also exhibits three additional modes corresponding to the segregated CuO phase. The observable blueshifts and broadening of the three modes of 50CuO/CeO_2−_ *_x_* relative to those of the CuO bulk counterpart can be attributed to the size effect, where the nanoscale CuO domains are directly interfaced with the Cu‐substituted surface of the CeO_2_ nanocrystals.^[^ ^14^, ^39^ ^]^ The relative intensity ratio between the D and F_2g_ modes (*I*_D_/*I*_F2g_), which is indicative of the oxygen defect concentration in ceria,^[^ ^40^ ^]^ also shows a volcano‐like relationship, increasing from CeO_2_ (*I*_D_/*I*_F2g_ ≈ 0) to 5CuO/CeO_2−_ *_x_* (*I*_D_/*I*_F2g_ = 0.22) and 10CuO/CeO_2−_ *_x_* (*I*_D_/*I*_F2g_ = 0.52), and then decreasing for 20CuO/CeO_2−_ *_x_* (*I*_D_/*I*_F2g_ = 0.29). Elevation of the Cu content in CuO/CeO_2−_ *_x_* would increase the concentration of the created oxygen defects, with the precondition that Cu is atomically dispersed above or substituted into the surface lattice of the underlying CeO_2_ support. Excess deposition of the bulk CuO phase can be adverse to the creation of oxygen defects. The dispersion of Cu was also analyzed using electron paramagnetic resonance (EPR). As shown in Figure S10b (Supporting Information), the intensity of the characteristic EPR peak corresponding to the isolated Cu^2+^ sites is prominent for 10CuO/CeO_2−_ *_x_* and 20CuO/CeO_2−_ *_x_*.^[^ ^41^ ^]^ In contrast, the peak intensity diminishes for 5CuO/CeO_2−_ *_x_* due to the low concentrations of the dispersed Cu^2+^ species. Magnetic coupling of the neighboring Cu^2+^ sites in 50CuO/CeO_2−_ *_x_* also observably quenches the EPR signal.^[^ ^42^ ^]^ Taking the Raman and EPR results together, concentration of the dispersed Cu(II) sites and the correlated oxygen defects both exhibit a volcano‐like relationship with regards to the Cu concentration. These data manifest the evolution of copper from isolated single atoms and dispersed clusters to segregated CuO domains supported on CeO_2_. It is thus inferred that enrichment of the dispersed Cu^2+^ sites facilitates creation of oxygen defects through charge compensation between the Cu^+^/Cu^2+^ and Ce^3+^/Ce^4+^ redox pairs. During the nanoparticle‐based atom‐trapping process, the restructuring behavior at the CuO/CeO_2_ interface produces atomically dispersed copper species, which also promotes creation of surface oxygen defects in the CeO_2_ nanocrystals.

### 2.3 CuO/CeO_2−__*x*_ Samples for Water–Gas Shift Reaction

**Figure 5:**
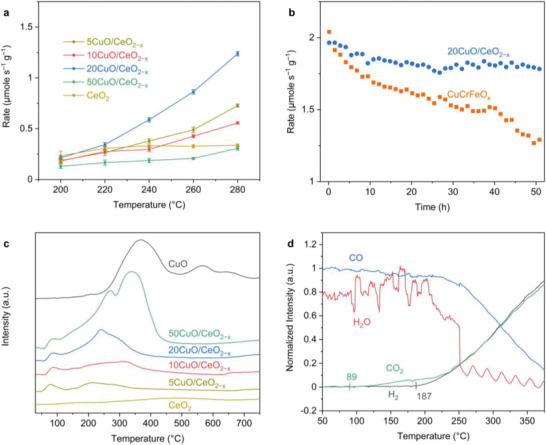
a) WGSR activity of the CuO/CeO_2−_ *_x_* samples at different temperatures. The reaction rates were measured within the kinetic zone. b) Stability test for the 20CuO/CeO_2−_ *_x_* and CuCrFeO*_x_* catalysts for WGSR at 350 °C. c) CO‐TPR profiles of the CuO/CeO_2−_ *_x_* samples. d) TPSR of 20CuO/CeO_2−_ *_x_* under WGSR conditions.

As a key industrial process for hydrogen production,^[^ ^43^, ^44^, ^45^ ^]^ WGSR relies on the efficient activation and conversion of CO and H_2_O molecules. For the commercial copper‐based catalysts, such as CuCrFeO*_x_*, the Cu/FeO*_x_* interfaces account for CO activation while H_2_O molecules are activated on the associated oxygen defect sites.^[^ ^46^ ^]^ Maximizing the dispersed Cu sites and the associated oxygen defects is desired, while excessive deposition of CuO would block the exposed active sites and consequently decrease the catalytic activities. Given the capability to controllably modulate the copper dispersion on ceria, we applied the atom‐trapped CuO/CeO_2−_ *_x_* samples for WGSR. As displayed in **Figure** **5**a; and Figure S11a,b (Supporting Information), 20CuO/CeO_2−_ *_x_* exhibited the highest catalytic activity for WGSR and reached 1.24 µmol s^−1^ g^−1^ at 280 °C, observably higher than that for 5CuO/CeO_2−_ *_x_* (0.73 µmol s^−1^ g^−1^), 10CuO/CeO_2−_ *_x_* (0.56 µmol s^−1^ g^−1^), and 50CuO/CeO_2−_ *_x_* (0.31 µmol s^−1^ g^−1^). As shown in Figure 5b, the optimal catalyst, 20CuO/CeO_2−_ *_x_*, also exhibited compelling stabilities during a 50 h stability test at 350 °C, which is superior to that of the CuCrFeO*_x_* catalyst prepared using the ammonia‐assisted coprecipitation method.^[^ ^47^ ^]^ The superior catalytic stability can be attributed to the strong chemical interactions between Cu and CeO_2_, which drives the decomposition and interfacial restructuring of the CeO_2_‐supported Cu nanoparticles.^[^ ^48^ ^]^

Temperature‐programmed reduction (TPR) experiments using CO as the probing molecule were performed to elucidate the influence of Cu dispersion on the redox properties of the CuO/CeO_2−_ *_x_* samples. As shown in Figure 5c, 5CuO/CeO_2−_ *_x_*, 10CuO/CeO_2−_ *_x_*, 20CuO/CeO_2−_ *_x_*, and 50CuO/CeO_2−_ *_x_* all exhibited the reduction peak at around 90 °C that corresponds to the reduction of the atomically‐dispersed Cu sites.^[^ ^31^ ^]^ The accompanied reduction peaks ranging from 100 to 400 °C are assigned to the aggregated CuO*_x_* domains with various sizes and geometries. Reduction of pure CeO_2_ and CuO were observed to take place at higher temperatures, thus suggesting construction of the CuO/CeO_2−_ *_x_* interface facilitates reduction of both the CuO and CeO_2_ components. Although creation of oxygen defects is favored by the higher Cu concentration (Table S5, Supporting Information), the active sites are blocked by the exsolution of the CuO phase upon a certain threshold, leading to the decrease of the catalytic activities (Figure 5a).

Temperature‐programmed surface reaction (TPSR) was conducted to monitor the evolution of the products (CO_2_ and H_2_) catalyzed by 20CuO/CeO_2−_ *_x_*, which afford mechanistic insights into the reaction pathways. Two mechanisms have been proposed for WGSR, the associative and the redox one.^[^ ^49^ ^]^ In the associative mechanism, the reaction between CO and H_2_O produces associated reaction intermediates such as surface formate, which decompose under reaction conditions and simultaneously releases CO_2_ and H_2_. In the redox mechanism, on the other hand, CO and H_2_O are separately activated, causing sequential production of CO_2_ and H_2_. We used 20CuO/CeO_2−_ *_x_* to catalyze WGSR as well as decomposition of formic acid (HCOOH), which has been identified as an intermediate species for WGSR. ^[^ ^49^ ^]^ As shown in Figure S11c (Supporting Information), decomposition of HCOOH results in a monotonic and similar evolution of CO_2_ and H_2_ starting at ≈150 °C, as evidenced by the overlap of the mass spectrometry signals of CO_2_ and H_2_. In contrast, the TPSR experiment of 20CuO/CeO_2−_ *_x_* under the WGSR conditions shows that CO_2_ and H_2_ are yielded at ≈89 and ≈187 °C, respectively (Figure 5d). This indicates that the WGSR catalyzed by 20CuO/CeO_2−_ *_x_* follows the redox pathway, where CO and H_2_O are individually activated on the dispersed Cu sites and the associated oxygen defects, respectively.^[^ ^35^ ^]^ The nanocrystal‐based atom‐trapping strategy enables controlled tuning of the exposed copper species as well as the nearby surface oxygen defects, and the two optimized subprocesses bring enhanced catalytic performance. As shown in Figure 3d, the 20CuO/CeO_2−_ *_x_* catalyst affords ample dispersed copper sites in the form of surface‐confined clustered CuO*_x_* domains, which effectively promote CO adsorption and activation.^[^ ^35^ ^]^ Fewer reactive copper sites, which are mostly atomically dispersed on the surface of the CeO_2_ domains, are formed for the 5CuO/CeO_2−_ *_x_* and 10CuO/CeO_2−_ *_x_* samples. On the other hand, further increasing the copper content bring excess CuO deposition on CeO_2_ (50CuO/CeO_2−x_), which blocks the reactive copper sites as well as the associated oxygen defect sites, and consequently lowered the WGSR reactivity. We also found that aging could measurably enhance the WGSR activity of the 20CuO/CeO_2−_ *_x_* catalyst (Figure S11d, Supporting Information). The adsorption of H_2_O and CO molecules could fine‐tune the surface structures, where the dynamic restructuring behavior under reaction conditions may expose the blocked active sites and consequently improve the catalytic conversions.

## 3 Conclusion

In summary, we have reported a nanocrystal‐based atom‐trapping strategy to access Cu‐CeO_2_ nanostructures with atomic precisions. The strong interactions between the Cu and CeO_2_ nanocrystals trigger the interfacial restructuring, where Cu become dispersed and stabilized on the CeO_2_ surface. Guided by this strategy, we were able to modulate the dispersion of copper species on ceria for catalytic applications. The optimized 20CuO/CeO_2−_ *_x_* brought enhanced activities and stabilities catalyzing WGSR, which are attributed to the optimized balance between the dispersed Cu sites for CO activation and the associated oxygen defects for H_2_O activation. From an application‐driven perspective, the demonstrated strategy can be further extended to the preparation of a wide scope of supported transition metal nanostructures. Combining the soft and solid‐state chemistry opens a new avenue toward building, understanding, and leveraging the dynamic behaviors of metal clusters anchored on reductive oxide supports. The insights provided here are also useful to elucidate metal–support interactions in complex systems, such as high‐entropy materials and single‐atom catalysts.^[^ ^2^, ^50^ ^]^

## 4 Experimental Section

### Chemicals

Cerium(III) nitrate hexahydrate (Ce(NO_3_)_3_·6H_2_O, 99% trace metals basis), copper(II) nitrate trihydrate (Cu(NO_3_)_2_·3H_2_O, purum p.a., 98.0–103% (RT)), copper(I) acetate (CuAc, 97%), oleylamine (technical grade, 70%), 1‐octadecene (technical grade, 90%), trioctylamine (98%), tetradecylphosphonic acid (TDPA, 98%), and white quartz (SiO_2_, ≥99.995%, trace metals basis) were purchased from Sigma‐Aldrich. Solvents, including hexane, ethanol, acetone, and chloroform were of analytical grade. All chemicals were used as received without further purification.

### Colloidal Synthesis of CeO_2_ and CuCeO_2−_ *_x_* Nanocrystals

Colloidal CeO_2_ and CuCeO_2−_ *_x_* nanocrystals were synthesized via modifying the method reported by Lee et al.^[^ ^21^ ^]^ For the preparation of the CeO_2_ nanocrystals, 868 mg of Ce(NO_3_)_3_·6H_2_O (2 mmol), 13 mL 1‐octadecene (40 mmol), and 2 mL oleylamine (6 mmol) were added to a 100 mL three‐neck flask at room temperature, and stirred under argon at 80 °C for 30 min to form a homogeneous solution. The mixture was then heated to 220 °C under argon. After 2 h, the reaction was stopped with the heating mantle removed. After cooling down to room temperature, the products were washed five times with a 1:1 ethanol/acetone mixture and stored in 10 mL of hexane for redispersion. For the preparation of the CuCeO_2−_ *_x_* nanocrystals, same synthetic protocol was employed with 10 at% of Cu(NO_3_)_2_·3H_2_O added.

### Colloidal Synthesis of Cu Nanocrystals

Colloidal Cu nanocrystals were synthesized via modifying a reported approach.^[^ ^20^ ^]^ 245.2 mg of CuAc (2 mmol), 278.4 mg of TDPA (1 mmol), and 20 mL trioctylamine (46 mmol) were added to a 100 mL three‐neck flask at room temperature, and degassed for 30 min under vacuum at 100 °C. Under argon, the solution mixture was heated to 180 °C and then to 270 °C, maintaining 30 min at each temperature. The reaction was then stopped with the heating mantle removed. After cooling down to 100 °C, ethanol was added, and the solution mixture was washed with chloroform and acetone three times. The obtained Cu nanoparticles were redispersed in 10 mL of hexane for further use.

### Fabrication of CuCeO_2−_ *_x_* and CuO/CeO_2−_ *_x_* Powder Samples

The copper‐doped ceria powder sample (CuCeO_2−_ *_x_*) was obtained through annealing in air at 500 °C for 4 h to remove the surface ligands. The CuO/CeO_2−_ *_x_* samples were prepared using a self‐assembly method.^[^ ^22^, ^23^ ^]^ Stoichiometric amounts of colloidal Cu and CeO_2_ nanocrystals and 1 wt% of squalane were added to 10 mL of hexane and stirred overnight. The solution mixture was then dried under ambient conditions, and evaporation of hexane triggered self‐assembly of the colloidal nanocrystals. The dried powder sample was annealed in nitrogen at 500 °C for 2 h to carbonize the surface‐capped ligands, ^[^ ^24^ ^]^ and then annealed in air at 500 °C for 4 h to remove the carbonized layer. The elemental composition was adjusted via tuning the added amount of the Cu and CeO_2_ colloidal nanocrystals. The obtained samples were labeled as 5CuO/CeO_2−x_, 10CuO/CeO_2−x_, 20CuO/CeO_2−x_, and 50CuO/CeO_2−x_, denoting the Cu content of 5, 10, 20, and 50 at%, respectively.

### Characterization

TEM images were acquired using an aberration‐corrected FEI Titan S 80‐300 STEM/TEM microscope equipped with a Gatan OneView camera at 300 kV. At least 100 particles were analyzed for the size‐distribution analysis. HAADF‐STEM images with STEM‐EDS element maps and HAABF‐STEM images were taken using a JEOL NEOARM operating at 200 kV. Powder XRD patterns were collected with a PANalytical Empyrean X‐ray diffractometer equipped with Cu K*α* radiation, with the operating voltage of 45 kV and current of 40 mA. Simulated XRD patterns were generated using the CrystalMaker/CrystalDiffract software package. Raman measurements with 532 nm laser were performed in a Renishaw inVia confocal microscope‐based Raman spectrometer. Inductively coupled plasma‐atomic emission spectroscopy (ICP‐AES) was performed using an Optima 2100 DV spectrometer (PerkinElmer Corporation). Thermal gravimetric analysis (TGA) data were obtained with TA Instrument TA Q50 under air. Nitrogen adsorption analysis was performed at 77 K with TriStar 3000, surface area was estimated using the Brunauer, Emmett, and Teller (BET) equation.^[^ ^51^ ^]^ XAS measurements were performed at the beamline 10‐ID‐B of the Advanced Photon Source at Argonne National Laboratory,^[^ ^52^ ^]^ and the data were processed and analyzed using the Athena and Artemis program of the IFEFFIT package based on FEFF 6.^[^ ^53^ ^]^ Neutron diffraction and pair distribution function (PDF) data were collected at the NOMAD beamline at the Spallation Neutron Source, Oak Ridge National Laboratory, and structure refinements were carried out using the TOPAS v6 software.^[^ ^54^ ^]^ More details over sample preparation and data collection and analysis for XAS and neutron diffraction are included in the Supporting Information.

### Catalytic Measurements

WGSR was performed using an AMI‐200 apparatus with a fixed‐bed reactor (U‐type quartz tube, 4 mm inner diameter) under atmospheric pressure. 20 mg of the CeO_2_‐based catalyst was diluted with 100 mg of white quartz, loaded into the reactor with quartz wool as support, pretreated with 10.0 vol% O_2_/Ar (30 mL min^−1^) at 500 °C for 1 h, and then reduced with 4.0 vol% H_2_/He for 2 h at 220 °C.^[^ ^35^ ^]^ The reaction was then tested at 200, 220, 240, 260, and 280 °C for 2 h at each temperature point, with a feed gas of 0.8 vol% CO/3.5 vol% H_2_O/He (17.6 mL min^−1^). For stability test, the catalyst was tested at 350 °C for 50 h with the same feed gas. The reactants and products were analyzed using an Agilent 7820 gas chromatograph (GC) equipped with a thermal conductivity detector and a flame ionization detector. For CO‐TPR experiments, 50 mg of the measured sample was loaded in a U‐type quartz with quartz wool as support, and pretreated using 10.0 vol% O_2_/Ar (30 mL min^−1^) at 500 °C for 1 h. After naturally cooling down to 30 °C, the sample was reduced in a flow of 1.0 vol% CO/He (30 mL min^−1^) from 30 to 800 °C with the ramp rate of 10 °C min^−1^. For TPSR experiments for WGSR and formic acid decomposition, 50 mg of 20CuO/CeO_2−_ *_x_* was loaded into the reactor with quartz wool as support, pretreated with 10.0 vol% O_2_/Ar (30 mL min^−1^) at 500 °C for 1 h, and then reduced with 4.0 vol% H_2_/He for 2 h at 220 °C. After cooling to 50 °C, the catalyst was heated to 500 °C with the ramp rate of 10 °C min, under the identical reaction condition (17.6 mL min^‒1^ of 0.8 vol% CO/3.5 vol% H_2_O/He) for the TPSR of WGSR, or accompanied by the introduction of formic acid (0.15 µL min^‒1^) using a Chemyx Nexus 3000 syringe pump with pure He as the feed gas. The outlet from the reactor for the CO‐TPR and TPSR experiments was analyzed using the mass spectrometer (Blazers Instruments, GSD‐300), and the species with the m/z value of 2, 18, 28, 29, 32, and 44 were recorded as H_2_, H_2_O, CO, HCOOH, O_2_, and CO_2_, respectively.

## Conflict of Interest

The authors declare no conflict of interest.

## Supporting information

Supporting Information

## Data Availability

The data that support the findings of this study are available from the corresponding author upon reasonable request.
