# Supplementary material for: Manipulating Copper Dispersion on Ceria for Enhanced Catalysis: A Nanocrystal‐Based Atom‐Trapping Strategy
Source: Adv Sci (Weinh). 2022 Jan 20;9(8):2104749. doi: 10.1002/advs.202104749 (PMC8922119; doi:10.1002/advs.202104749)
Supplement: Supplementary file 1 — Supporting Information [file ADVS-9-2104749-s001.pdf]

ADVANCED SCIENCE
Open Access

Supporting Information

for *Adv. Sci.*, DOI 10.1002/advs.202104749

Manipulating Copper Dispersion on Ceria for Enhanced Catalysis: A Nanocrystal-Based Atom-Trapping Strategy

*Yifan Sun, Felipe Polo-Garzon, Zhenghong Bao, Jisue Moon, Zhennan Huang, Hao Chen, Zitao Chen, Zhenzhen Yang, Miaofang Chi, Zili Wu, Jue Liu and Sheng Dai**

WILEY-VCH

ADVANCED SCIENCE
Open Access

Supporting Information

for *Adv. Sci.*, DOI: 10.1002/advs.202104749

Manipulating Copper Dispersion on Ceria for Enhanced Catalysis: A Nanocrystal-Based Atom-Trapping Strategy

*Yifan Sun, Felipe Polo-Garzon, Zhenghong Bao, Jisue Moon, Zhennan Huang, Hao Chen, Zitao Chen, Zhenzhen Yang, Miaofang Chi, Zili Wu, Jue Liu, Sheng Dai**

Supporting Information

Manipulating Copper Dispersion on Ceria for Enhanced Catalysis: A Nanocrystal-Based Atom-Trapping Strategy

Yifan Sun, Felipe Polo-Garzon, Zhenghong Bao, Jisue Moon, Zhennan Huang, Hao Chen, Zitao Chen, Zhenzhen Yang, Miaofang Chi, Zili Wu, Jue Liu, Sheng Dai*

Y. Sun, F. Polo-Garzon, Z. Bao, J. Moon, Z. Yang, Z. Wu, S. Dai
Chemical Sciences Division, Oak Ridge National Laboratory, Oak Ridge, TN 37831, USA
E-mail: dais@ornl.gov
Z. Huang, Z. Chen, M. Chi, Z. Wu
Center for Nanophase Materials Sciences, Oak Ridge National Laboratory, Oak Ridge, TN 37831, USA
H. Chen, S. Dai
Department of Chemistry, The University of Tennessee, Knoxville, TN 37996, USA
J. Liu
Neutron Scattering Division, Oak Ridge National Laboratory, Oak Ridge, TN 37831, USA

Supplementary Experimental

XAS measurement: XAS Spectra were collected at Ce L-III edge (eV) in transmission mode with vanadium foil as the reference. Cu K edge (eV) was measured in fluorescence mode with corresponding metal foils as the reference for energy calibration as well as data analysis. The X-ray beam was monochromatized by a Si (111) monochromator. The incident beam intensity (I_0), transmitted beam intensity (I_t), fluorescence intensity (I_f) and reference (I_{ref}) were all measured by 20 cm ionization chambers. All spectra were collected at room temperature. Samples were centered on the beam and 10 scans were collected for each sample. Data were rebinned with grids of 10 eV, 0.5 eV, and 0.05 eV for the pre-edge, XANES, EXAFS region, respectively. The adsorption energy (E_0) was determined based on the first derivative of the edge. Reference foil data were aligned to the first zero-crossing of the second derivative of the normalized μ(E) data, which was calibrated to the literature E_0 for the Cu edge (8978.9 eV). All data were initially fitted with k-weighing of 1, 2 and 3, and then finalized with k^2-weighting in R-space. A fit of the metal foil standard was used to determine S_0^2 for the samples. Structural parameters were determined by the fits including the degeneracy of the scattering path (N_{degen}), the change in R_{eff} (ΔR_i), the mean square relative displacement of the scattering element ($\Delta\sigma^2_i$), and the energy shift of the photoelectron (ΔE_0). For each fit, the number of independent points was not permitted to exceed 2/3 the number of variables, in keeping with the Nyquist criterion.[1]

Neutron total scattering measurements and PDF fitting: For the neutron diffraction measurement, 0.2 g CeO_2-based nanocrystals were loaded into 3 mm quartz capillaries. Four 24 min scans were collected for each sample and then summed together to improve the statistics of the data. Signal from the empty quartz capillary measurement was subtracted as background and the obtained data were normalized by the scattering intensity from a 6 mm vanadium rod corrected for attenuation effects. Small-box least square method was applied to analyze the neutron pair distribution function g(r) data in TOPAS version 6.[2] A Q_{max} cut-off of 30 $Å^{-1}$ was adopted for all four samples during Fourier transform of the S(Q) to the PDF g(r). No absorption correction was applied due to the small absorption cross sections associated with Ce and O atoms. The correction of incoherent inelastic scattering from H (nuclear recoil) was done using the add-hoc method, i.e., using the right side of the pseudo-Voigt function. To obtain the near absolute normalized data, Fourier filter was used to direct g(r) to zero for $r < 0.8$ Å. Scale factors were refined to near unit (ranging from 1.02 to 1.04) for the final reduced g(r), confirming the data were appropriately normalized. For PDF data

analysis, the instrument constants Q_{broad} and Q_{damp} were determined from the refinement of a Ni standard to be 0.24 $Å^{-1}$ and 0.22 $Å^{-1}$, separately.[3] The analytical spherical shape function (or envelope function) was used to quantify the shape and size effect for the CeO_2 and CuO nanoscale domains.[4]

Supplementary Tables

Table S1 Cu content in the CuO/CeO_{2-x} samples quantified using ICP-AES.

Sample	Cu Percentage / %
**5CuO/CeO$_{2-x}$**	4.5
**10CuO/CeO$_{2-x}$**	8.8
**20CuO/CeO$_{2-x}$**	15.6
**50CuO/CeO$_{2-x}$**	44.9

Table S2 Fitting parameters of the Cu K-edge EXAFS for $CuCeO_{2-x}$, $10CuO/CeO_{2-x}$ and $20CuO/CeO_{2-x}$.

				**Cu−O**			**Cu−Cu in CuO**	
**Sample**	**Δ E$_0$ / eV**	**R-factor**	**CN**	**R / Å**	**Δσ^2 / Å^2**	**CN**	**R / Å**	**Δσ^2 / Å^2**
**CuCeO$_{2-x}$**	−4.9 (0.907)	0.0106	3.3 (0.5)	1.942 (0.007)	0.004 (0.0002)			
**10CuO/CeO$_{2-x}$**	−3.37 (0.917)	0.00517	3.1 (0.2)	1.948 (0.005)	0.004 (0.0007)			
**20CuO/CeO$_{2-x}$**	−1.53 (0.906)	0.0206	3.1 (0.5)	1.952 (0.0058)	0.003 (0.0001)	0.9 (0.2)	2.927 (0.01)	0.007 (0.0001)

Table S3 Short-range (0.4-10 Å) fitting results of the neutron pair distribution function g(r) for the CeO_2 and CuO/CeO_{2-x} samples. For $20CuO/CeO_{2-x}$, PDF fitting using the tri-phase model (O−H, nanosized CuO and CeO_2) significantly enhances the fitting quality compared with that solely using the CeO_2 model.

**Sample**	**Lattice Parameter a / Å**	**R_{WP} / %**
**CeO_2**	5.4201(6)	7.7
**$10CuO/CeO_{2-x}$**	5.4204(10)	13.8
**$20CuO/CeO_{2-x}$**	5.4241(33)	33.6
**$20CuO/CeO_{2-x}$ (Tri-phase)**	5.4255(9)	7.3

Table S4 Intermediate-range (0.4-40 Å) fitting results of the neutron pair distribution function g(r) for the CeO_2 and CuO/CeO_{2-x} samples.

**Sample**	**Lattice Parameter a / Å**	**Coherent Crystallite Size d / Å**	**R_{WP} / %**
**CeO_2**	5.4182(1)	80.2(9)	4.4
**$10CuO/CeO_{2-x}$**	5.4190(4)	54.9(8)	7.6
**$20CuO/CeO_{2-x}$**	5.4209(11)	55.4(2)	19.6

Table S5 CO consumption amount quantified using the CO-TPD data in Figure 5c.

**Sample**	**CO Consumption / mmol g^{-1}**
**CeO_2**	1050
**$5CuO/CeO_{2-x}$**	1140
**$10CuO/CeO_{2-x}$**	1160
**$20CuO/CeO_{2-x}$**	1170
**$50CuO/CeO_{2-x}$**	2510
**CuO**	1960

Supplementary Figures

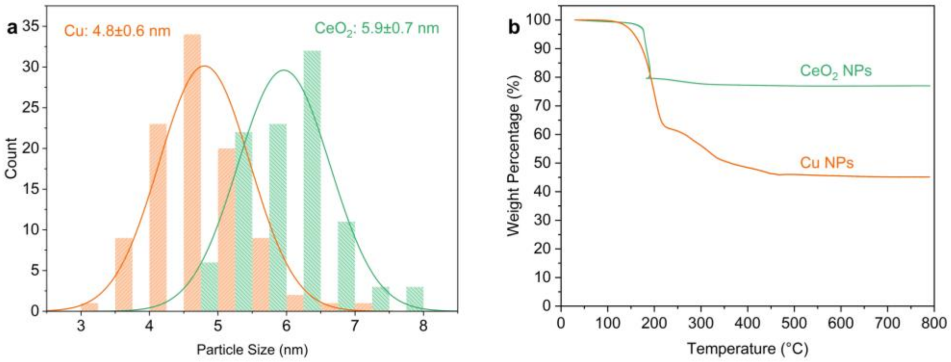

Figure S1. (a) Size distribution histogram and (b) TGA data for the colloidal Cu and CeO_2 nanocrystals. At least 100 particles were analyzed for the size-distribution analysis.

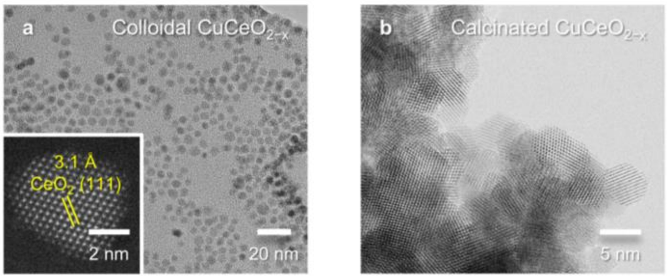

Figure S2. (a) TEM image of the colloidal $CuCeO_{2-x}$ nanoparticles with the high-resolution HAADF-STEM image in the inset. (b) HAABF-STEM image of the calcinated $CuCeO_{2-x}$ nanoparticles.

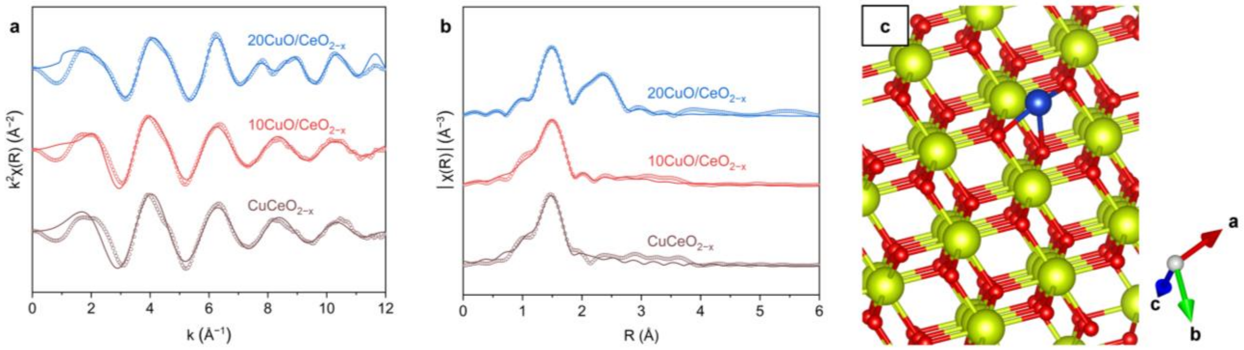

Figure S3. Cu K-edge EXAFS spectra in (a) k-space and (b) R-space for $CuCeO_{2-x}$, $10CuO/CeO_{2-x}$ and $20CuO/CeO_{2-x}$, with the fitting parameters listed in Table S2. (c) Schematic showing the Cu_1O_3 geometry on the CeO_2 {111} surface. Cu, Ce and O atoms are represented by blue, yellow and red spheres.

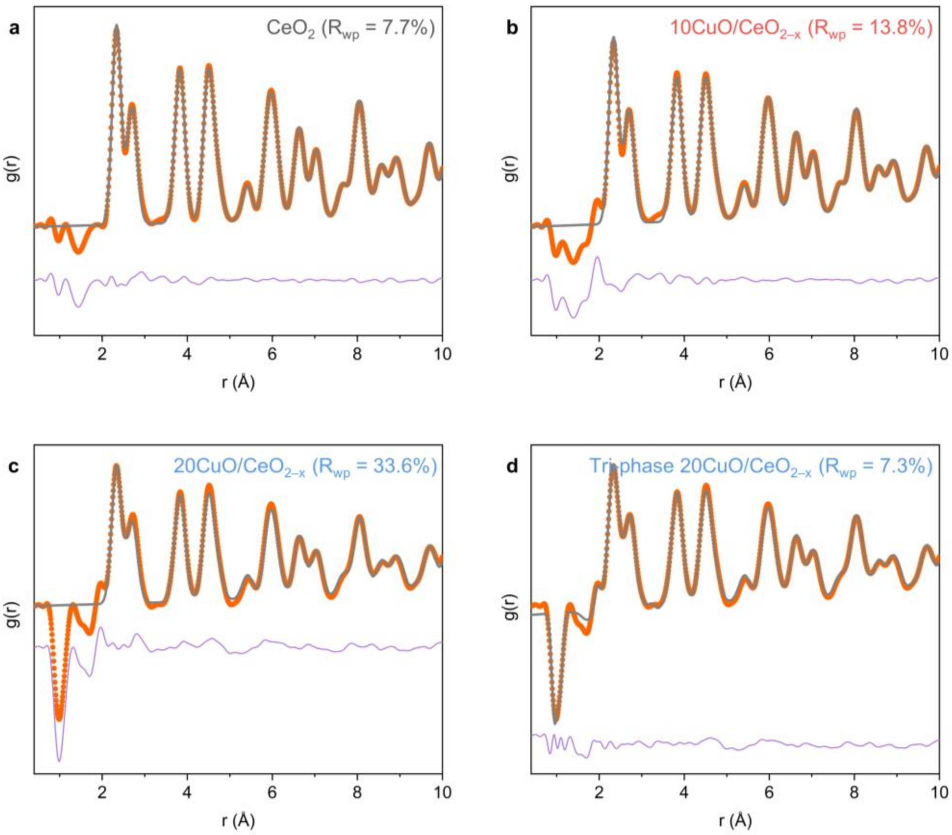

Figure S4. Short-range (0.4–10 Å) g(r) analysis of (a) CeO_2, (b) 10CuO/CeO_{2-x}, 20CuO/CeO_{2-x} using the (c) single-phase and (d) tri-phase model. The fitting results are summarized in Table S3. For 20CuO/CeO_{2-x}, PDF fitting using the tri-phase model (O−H, nanosized CuO and CeO_2) significantly enhances the fitting compared with that solely using the CeO_2 model.

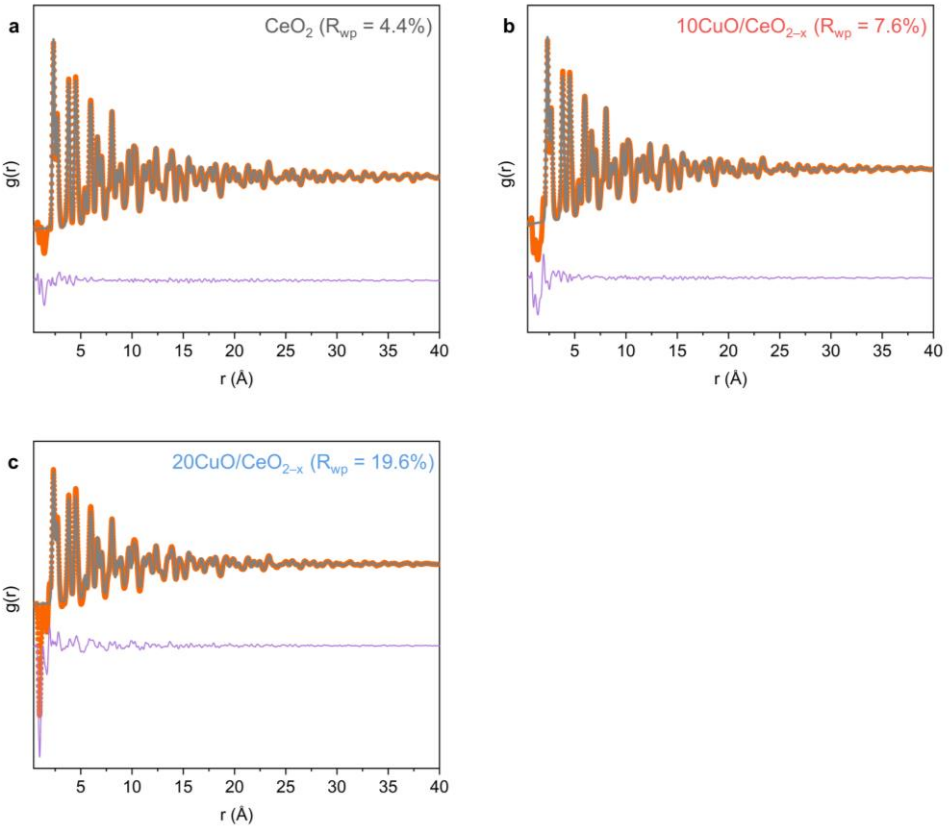

Figure S5. Intermediate-range (0.4–10 Å) g(r) analysis of the CeO_2 and CuO/CeO_{2-x} samples. The corresponding fitting results are summarized in Table S4.

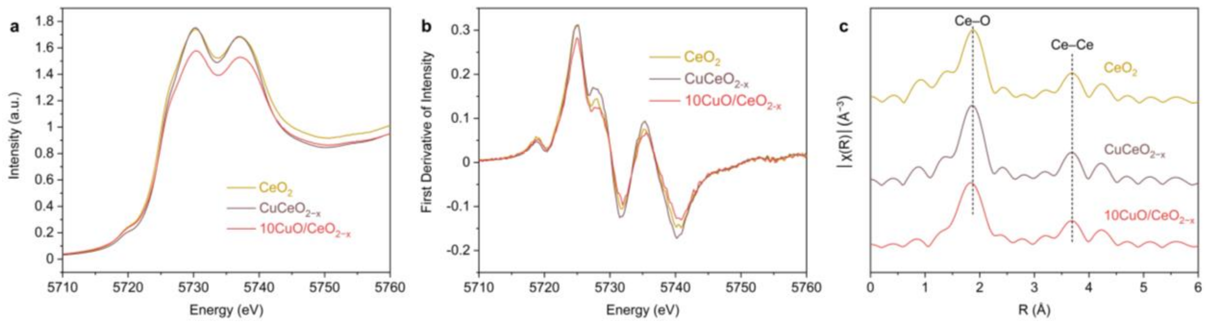

Figure S6. (a) Ce L_{III}-edge XAS spectra, (b) the corresponding first derivative and (c) EXAFS of $CuCeO_{2-x}$, $10CuO/CeO_{2-x}$ and CeO_2.

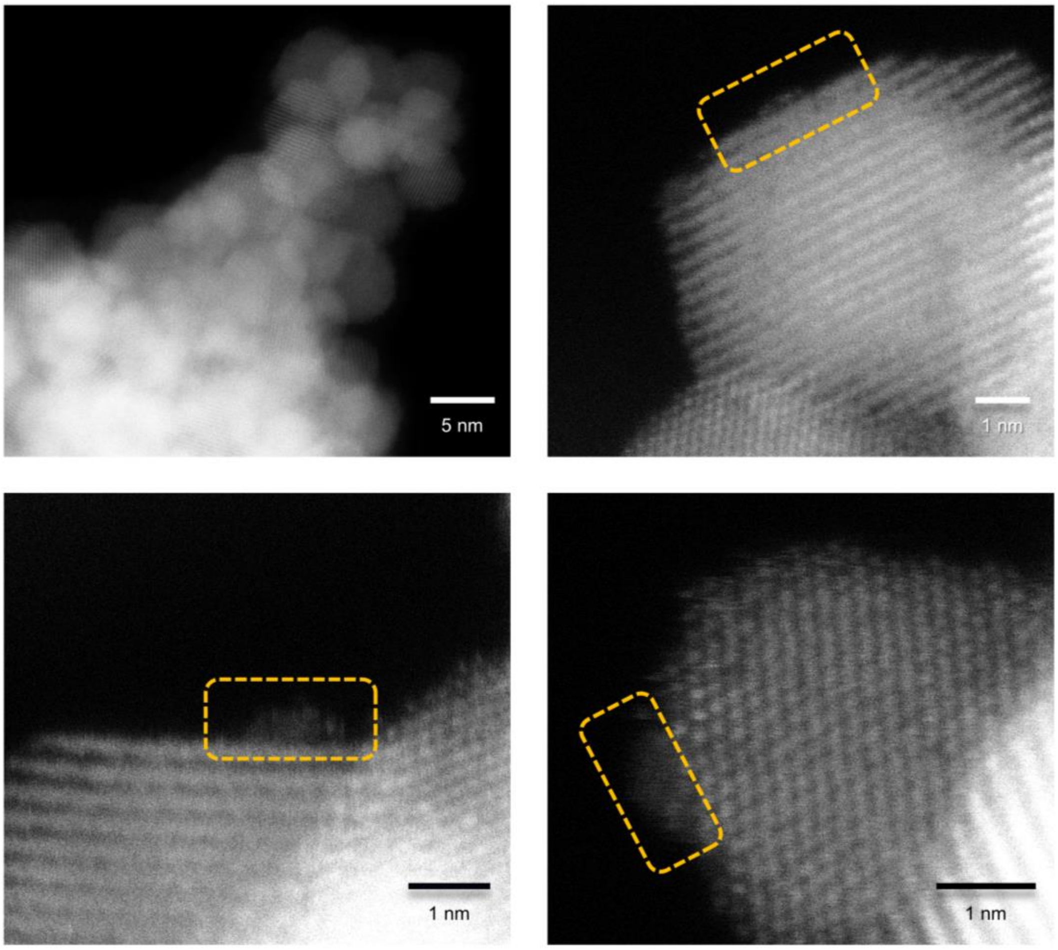

Figure S7. High-resolution HAADF-STEM images of 20CuO/CeO_{2-x}, where the atomic CuO_x layers are highlighted using dotted box in light orange.

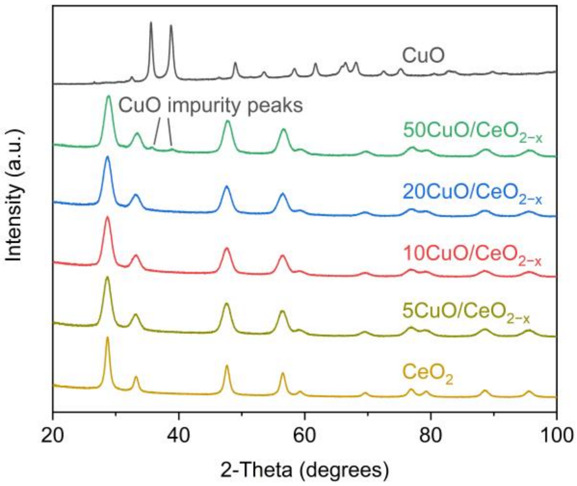

Figure S8. XRD patterns the CeO_2, CuO/CeO_{2-x} and CuO samples prepared using the atom-trapping strategy.

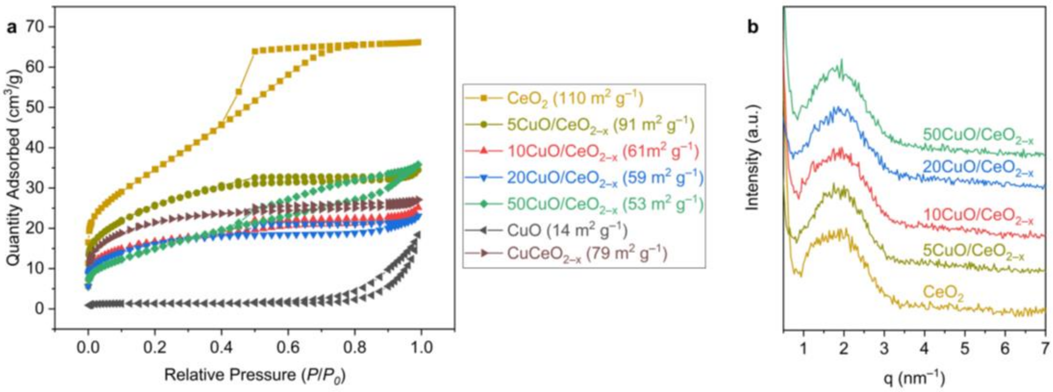

Figure S9. (a) N_2 adsorption isotherms and (b) SAS data of the CuO/CeO_{2-x} samples.

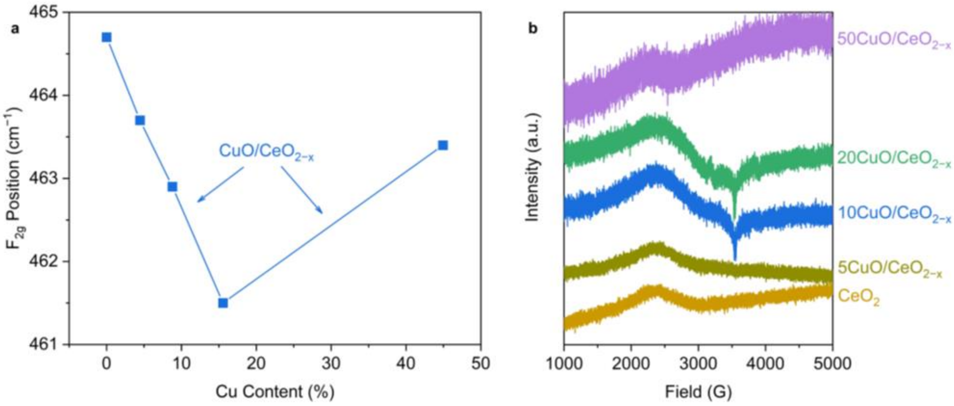

Figure S10. (a) Plot of F_{2g} position vs. Cu content in the CuO/CeO_{2-x} samples, showing the composition-dependent Raman frequency of the F_{2g} peak. (b) EPR results of the CuO/CeO_{2-x} samples.

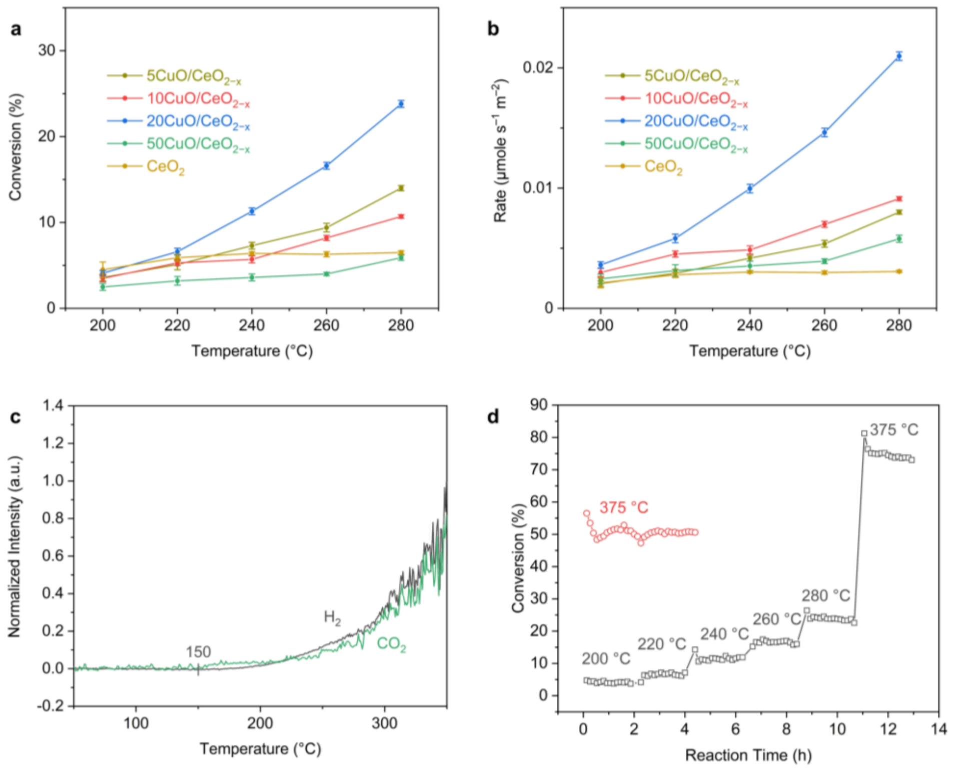

Figure S11. CuO/CeO_{2-x}-catalyzed WGSR activities in (a) percentage conversion and (b) rate per surface area. (c) TPSR showing the thermal decomposition of HCOOH on $20CuO/CeO_{2-x}$, where H_2 and CO_2 are simultaneously produced. (d) Comparison of the WGSR activity using the $20CuO/CeO_{2-x}$ catalyst with (red) and without aging (black) under the WGSR conditions.

References

[1] S. Calvin, *XAFS for Everyone*; CRC press, **2013**.

[2] A. A. Coelho, P. A. Chater, A. Kern, *J. Appl. Crystallogr.* **2015**, *48*, 869.

[3] C. L. Farrow, P. Juhas, J. W. Liu, D. Bryndin, E. S. Božin, J. Bloch, T. Proffen, S. J. L. Billinge, *J. Phys. Condens. Matter* **2007**, *19*, 335219.

[4] J. Liu, D. Olds, R. Peng, L. Yu, G. S. Foo, S. Qian, J. Keum, B. S. Guiton, Z. Wu, K. Page, *Chem. Mater.* **2017**, *29*, 5591.
